# Clinical outcomes of extracorporeal membrane oxygenation in acute traumatic lung injury: a retrospective study

**DOI:** 10.1186/s13049-020-00733-w

**Published:** 2020-05-24

**Authors:** Hong Kyu Lee, Hyoung Soo Kim, Sang Ook Ha, Sunghoon Park, Hee Sung Lee, Soo Kyung Lee, Sun Hee Lee

**Affiliations:** 1 Department of Thoracic and Cardiovascular Surgery, Hallym University Sacred Heart Hospital, Hallym University Medical Center, Gwanpyeong-ro 170 beon-gil 22, Dongan-gu, Anyang-si, Gyeonggi-do 14068 South Korea; 2 Department of Emergency Medicine, Hallym University Sacred Heart Hospital, Hallym University Medical Center, Anyang-si, Gyeonggi-do South Korea; 3 Division of Pulmonary, Allergy, and Critical Care Medicine, Hallym University Sacred Heart Hospital, Hallym University Medical Center, Anyang-si, Gyeonggi-do South Korea; 4 Department of Thoracic and Cardiovascular Surgery, Hallym University Dongtan Sacred Heart Hospital, Hallym University Medical Center, Hwaseong-si, Gyeonggi-do South Korea; 5 Department of Anesthesiology and Pain Medicine, Hallym University Sacred Heart Hospital, Hallym University Medical Center, Anyang-si, Gyeonggi-do South Korea

**Keywords:** Acute respiratory distress syndrome, Extracorporeal membrane oxygenation, Trauma, Traumatic lung injury

## Abstract

**Background:**

Therapeutic extracorporeal membrane oxygenation (ECMO) is a challenging procedure in patients who have experienced severe trauma. Particularly, patients with traumatic lung injury and posttraumatic acute respiratory distress syndrome (ARDS) have a high risk of bleeding during this procedure. This study aimed to determine the safety and feasibility of ECMO in patients with traumatic ARDS.

**Methods:**

We retrospectively reviewed medical records and investigated the clinical outcomes of ECMO in 42 patients with traumatic ARDS, among whom near-drowning (42.9%) was the most frequent cause of injury.

**Results:**

Thirty-four of 42 patients (81%) survived and were discharged after a median hospital stay of 23 days. A multivariate analysis identified a lactate level (odds ratio: 1.493, 95% confidence interval: 1.060–2.103, *P* = 0.022) and veno-venous (VV) ECMO (odds ratio: 0.075, 95% confidence interval: 0.006–0.901, *P* = 0.041) as favorable independent predictors of survival in patients with traumatic ARDS who underwent ECMO. The optimal cut off value for pre-ECMO lactate level was 10.5 mmol/L (area under the curve = 0.929, *P* = 0.001*).* In Kaplan-Meier analysis, the survival rate at hospital discharge was significant higher among the patients with a pre-ECMO lactate level of 10.5 mmol/L or less compared with patients with pre-ECMO lactate level greater than 10.5 mmol/L (93.8% versus 40.0%, respectively; *P =* 0.01).

**Conclusions:**

ECMO yielded excellent survival outcomes, particularly in patients with low pre-treatment lactate levels who received VV ECMO. Therefore, ECMO appears safe and highly feasible in a carefully selected population of trauma patients.

## Background

Pneumonia, sepsis and trauma are among the most common causes of acute lung injury or acute respiratory distress syndrome (ARDS) [1]. Severe trauma is generally accompanied by various injuries, including traumatic lung injury, leading to posttraumatic ARDS in some patients [2]. Major trauma-induced lung injury is associated with mortality rates as high as 50–80% due to direct consequences of the injury or secondary effects, such as hypoxemia and respiratory acidosis, including the injured brain [3]. Previous reports have described the efficacy of ECMO support in adult trauma patients with ARDS [4] and trauma patients with severe lung injury [5]. Traditionally, trauma patients have a high risk of bleeding and thus have not been considered suitable candidates for ECMO due to the limited experience of the attending medical practitioners. Accordingly, the utility of ECMO in trauma patients remains unclear.

In this retrospective study, we aimed to evaluate our experiences with ECMO support in patients with life-threatening acute traumatic lung injury and their clinical outcomes, and to identify the significant factors associated with survival outcomes.

## Methods

**Fig. 1:**
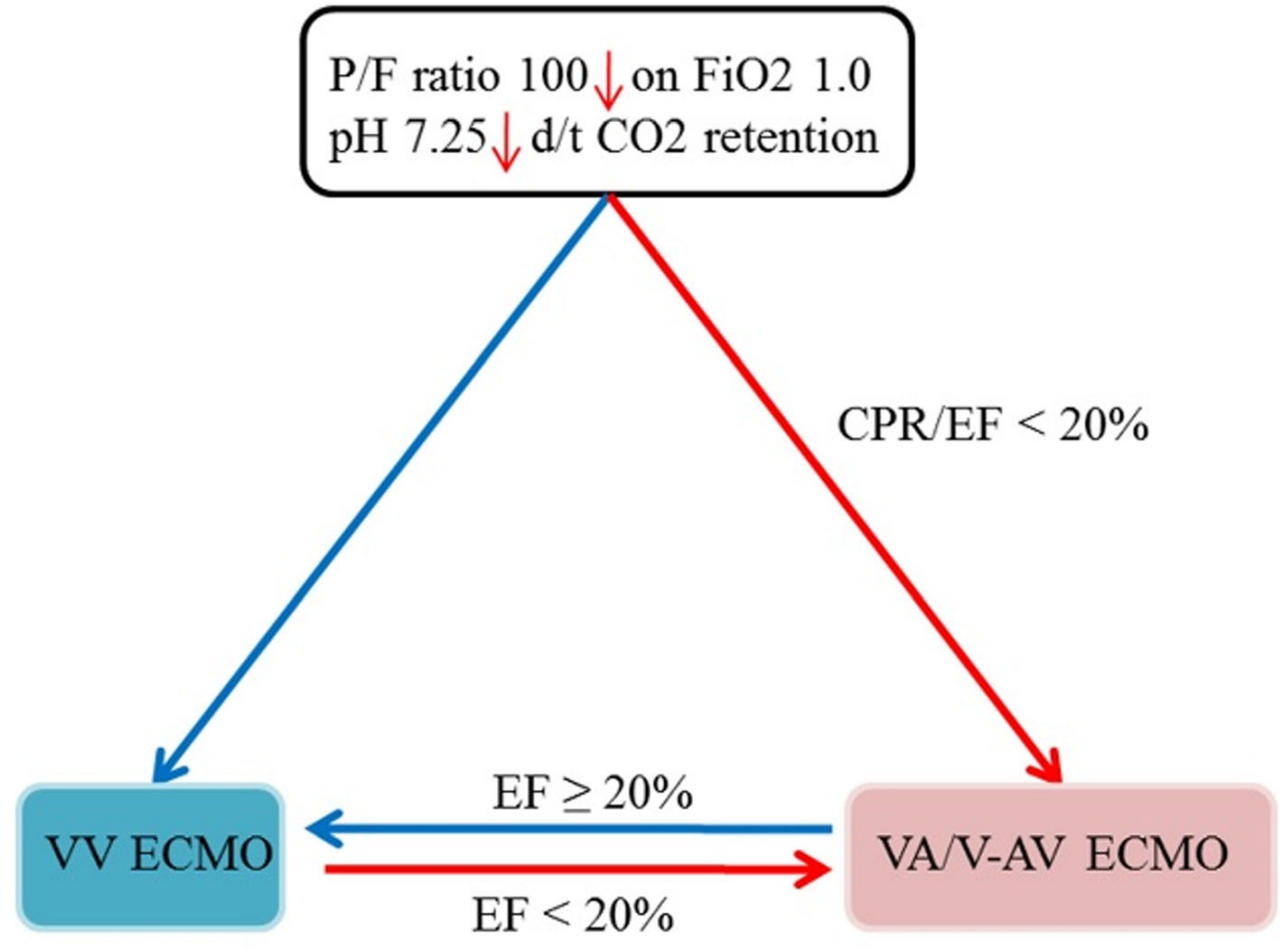
Schematic of the principle of ECMO configurations. Abbreviations: ECMO, extracorporeal membrane oxygenation; P/F, partial arterial oxygen pressure to fractional inspired oxygen concentration; CPR, cardiopulmonary resuscitation; EF, ejection fraction; VV, venovenous; VA. venoarterial; V-AV, venoarteriovenous

We retrospectively reviewed the data from 42 consecutive patients who received ECMO respiratory support for severe trauma-induced acute respiratory failure between January 2007 and December 2018. Since the hospital serves as a regional emergency medical center, all major trauma patients were admitted to the emergency department by Emergency Medical Service. Of the total 42, 10 received ECMO during intensive care in intensive care units (ICUs), while 32 received ECMO at emergency centers before ICU admission. The indications for veno-venous (VV) ECMO were partial arterial oxygen pressure to fractional inspired oxygen concentration (PaO2/FiO2) ratio < 100 with FiO2 of 1.0 and severe hypercapnia (pH < 7.25) with unstable hemodynamics, despite appropriate conventional ARDS treatment [6]. Venoarterial (VA) or venoarteriovenous (V-AV) ECMO was applied to patients whose ejection fractions on 2D echocardiograms were < 20% (Fig. 1). Indications for VA ECMO included coexistent shock (i.e., systolic blood pressure < 80 mmHg) with hypoxemia or hypercapnia despite fluid resuscitation and vasopressor support. Patients predicted to require high-dose vasopressors (i.e., norepinephrine > 0.5 μg/kg/min) during VV ECMO also received V-AV ECMO (Fig. 1). In V-AV ECMO, we inserted the additional arterial cannulae into the femoral artery and connected venous perfusion cannulae in right atrium using Y-connector. We controlled the degree of clamping of the femoral artery cannulae while monitoring the flowmeter for determining the amount of oxygenated perfusion flow and the systemic circulatory support. ECMO was contraindicated for patients with uncontrolled traumatic bleeding, severe hypoxemic brain injury, untreatable cancer and severe disability. Study approval was received from Hallym University Sacred Heart Hospital Institutional Review Board (IRB No. 2019–05–029-001), which waived the requirement for informed consent due to the retrospective design of the study.

### ECMO technique and management

We used two types of centrifugal pumps for ECMO. Until May 2010, the Capiox Emergency Bypass System® (Terumo, Inc., Tokyo, Japan) was used at our institution. However, since June 2010, the Centrifugal Rotaflow Pump® (Maquet Inc., Hirrlingen, Germany) has been used for most patients [6]. VV ECMO or VA ECMO were cannulated percutaneously using the Seldinger technique under fluoroscopic guidance in a cardiac catheterization laboratory or hybrid operating room in the emergency department. Arterial cannulae (17–21 Fr) and venous cannulae (17–28 Fr; DLP and Bio-Medicus, Medtronic, Minneapolis, MN, USA; RMI, Edward’s Lifesciences LLC, Irvine, CA, USA) were used depending on the patient’s size. The initial gas and blood flow rates were 4–6 and 3–5 L/min, respectively, to maintain an oxygen saturation (SaO2) > 90%. During ECMO support, heparin or nafamostat mesilate (SK Chemicals Life Science Biz., Seoul, Korea; licensed by Torii Pharmaceutical, Tokyo, Japan) was used for anticoagulation, with a target activated partial thromboplastin time (aPTT) of 60–80 s [7]. Anticoagulation was usually initiated after confirmed bleeding control was achieved for 48 h in patients with a high risk of bleeding or those who underwent surgery. The following mechanical ventilator settings were applied initially: tidal volume, 4–6 mL/kg; PEEP, 4–8 cm H2O; respiration rate, 10/min and FiO2, 0.21–0.6. Blood transfusions were administered as packed red blood cells (RBCs) with hemoglobin concentration < 8–10 g/dL, fresh frozen plasma (FFP) with an international normalized ratio (INR) > 2.0, platelet concentrate with a count of < 50,000/μL, or cryoprecipitate with a fibrinogen concentration of < 150 mg/dl.

ECMO weaning was considered when hemodynamic stability and pulmonary improvement were evident, and implemented once cardiac pulsatility and contractility had improved (left ventricular ejection fraction ≥30–40%). The pump flow and sweep gas were initially tapered to 2 and 0 L/min, respectively. For VA ECMO weaning, the pump flow was decreased by 1 L/min for 24 h. Decannulation was performed after the patient remained stable for 24 h. Weaning was considered successful when the patient remained stable for 48 h after ECMO removal.

### Data collection and statistical analysis

For each patient, we recorded demographics (age, sex, and body mass index), comorbidities, causes of injury, the Inotropic Score (IS), the Vasoactive-Inotropic Score (VIS), illness severity measure scores [Simplified Acute Physiology Score II (SAPSII) and Sequential Organ Failure Assessment (SOFA)] and laboratory data obtained immediately before initiating ECMO support. The IS have been used in clinical research as measures of illness severity in patients undergoing congenital heart surgery [8]. Several studies showed that the VIS, which indicates the amount of cardiovascular support by various inotropes or vasopressors, was independently predictive of clinical outcomes in patients who underwent cardiac surgery and in sepsis patiets [9]. The SAPS II and SOFA are severity scores and mortality estimation tools that were developed for patients in medical or surgical ICUs [10]. We also recorded the ECMO durations and weaning rates, transfusion and complication rates and all outcomes. All data obtained during ECMO support were compared between survivors and non-survivors. All results are presented as numbers with percentages for categorical variables, and as medians with interquartile ranges (IQRs) for continuous variables. The Mann–Whitney U test or repeated-measure analysis of variance (ANOVA) was used to compare continuous variables, whereas the chi-squared or Fisher’s exact test was used to compare categorical variables.

Univariate and multivariate logistic regression analyses were performed to identify independent risk factors for hospital mortality among the pre-ECMO variables and treatment variables during ECMO. Covariates identified as significant (*P* < 0.05) in the univariate analysis were included in the multivariate analysis. We further conducted a receiver operating characteristic curve analysis to identify the relevant cut-off values of independent pre-ECMO variables. Groups stratified using a pre-ECMO lactate concentration cut-off of 10.5 mmol/L were then subjected to a survival analysis based on the Kaplan–Meier method. IBM SPSS version 24.0 software for Windows (IBM Corp., Armonk, NY, USA) was used for all statistical analyses.

## Results

### Baseline characteristics

The baseline characteristics of the 42 patients who underwent ECMO following an acute traumatic lung injury are shown in Table 1. Thirty-seven (88.1%) of these patients were male, and the median age was 41 years. Acute traumatic lung injury was most frequently attributed to near drowning (*n* = 18, 42.9%), traffic accidents (*n* = 9, 21.4%), crush injuries to the chest (*n* = 5, 11.9%), falls from significant heights (*n* = 4, 9.5%), pulmonary hemorrhage due to stabbing (*n* = 2, 4.8%) or gunshot wounds to the chest (n = 1, 2.4%), intoxication-induced pneumonitis (n = 2, 4.8%) and hanging (n = 1, 2.4%). Most of the associated injuries occurred in the thorax. Seventeen (40.5%) patients required surgical treatment, of whom 7 (16.6%), 5 (11.9%), 4 (9.5%) and 1 (2.4%) underwent thoracic, skeletal, abdominal, or neurosurgery, respectively. Before ECMO, the mean IS, VIS, SOFA score and SAPSII were 4.28, 61.09, 11.50 and 54.57, respectively. The pre-ECMO median pH, PaO2, PCO2 and PaO2/FiO2 were 7.19, 60.5 mmHg, 47.0 mmHg and 61.5, respectively (Table 2).

**Table 1:** Comparison of baseline characteristics in survivors and non-survivors

Pre-ECMO	Total	Survivors	Non-survivors	*P*
Variables	*n* = 42 (%)	*n* = 34 (81%)	*n* = 8 (19%)	
Sex				0.564
Male	37 (88.1)	29 (69)	8 (19)	
Female	5 (11.9)	5 (11.9)	0 (0)	
Age (years), median (IQR)	41 (18.75–52.75)	39 (18.75–48.0)	49.5 (24.5–59.0)	0.378
BMI (kg/m^2^), median (IQR)	22.3 (20.7–24.0)	22.5 (21.1–24.5)	21.1(16.7–21.9)	0.277
Comorbidity				
Diabetes	5 (11.9)	5 (11.9)	0 (0)	0.327
Hypertension	6 (14.3)	5 (11.9)	1 (2.4)	0.681
Liver cirrhosis	2 (4.8)	0 (0)	2 (4.8)	0.033*
Asthma	1 (2.4)	0 (0)	1 (2.4)	0.190
CPR	21 (50)	16 (38.1)	5 (11.9)	0.348
MV before ECMO, median days (IQR)	1 (1–2.25)	1 (1–1.25)	1 (1–5.25)	0.270
Door to ECMO, median hours (IQR)	3.5 (2–81)	3.5 (2.2–81)	e	0.328
ECMO within 24 h	28 (67)	23 (58)	5 (11.9)	0.543
Surgical procedures	17 (40.5)	13 (31)	4 (9.5)	0.411
e Surgical procedures with ECMO	6 (14.3)	6 (14.3)	0 (0)	
Thoracic/abdominal/neuro/skeletal	4 /0 /0 /2	4 /0 /0 /2	0 /0 /0 /0	
ECMO after surgical procedures	11 (26.2)	7 (16.7)	4 (9.5)	
Thoracic/abdominal/neuro/skeletal	3 /4 /1 /3	3 /1 /1 /2	0 /3 /0 /1	
IS, mean (range)	4.28 (0–44.44)	4.64 (0–44.44)	2.75 (0–22.0)	0.680
VIS, mean (range)	61.09 (0–446.0)	35.68 (0–285.0)	169.09 (0–446.0)	0.001*
SOFA score, mean (range)	11.50 (4.0–19.0)	11.23 (4.0–16.0)	12.62 (8.0–19.0)	0.289
SAPSII score, mean (range)	54.57 (26.0–86.0)	51.08 (26.0–84.0)	69.37 (42.0–86.0)	0.010*
Trauma types				0.569
Car accident	9 (21.4)	8 (19)	1 (2.4)	
Near drowning	18 (42.9)	15 (35.7)	3 (7.1)	
Gunshot wound	1 (2.4)	1 (2.4)	0 (0)	
Intoxication	2 (4.8)	1 (2.4)	1 (2.4)	
Crushing injury	5 (11.9)	3 (7.1)	2 (4.8)	
Fall down	4 (9.5)	4 (9.5)	0 (0)	
Hanging	1 (2.4)	1 (2.4)	0 (0)	
Stabbed wound	2 (4.8)	1 (2.4)	1 (2.4)	
Associated injury				0.042*
Thorax	20 (47.6)	17 (40.5)	3 (7.1)	
T + head	5 (11.9)	4 (9.5)	1 (2.4)	
T + abdomen	1 (2.4)	0 (0)	1 (2.4)	
T + H + A + pelvis	1 (2.4)	1 (2.4)	0 (0)	
T + H + A + P + MSI	2 (4.8)	2 (4.8)	0 (0)	
T + H + A + MSI	3 (7.1)	3 (7.1)	0 (0)	
T + H + MSI	1 (2.4)	1 (2.4)	0 (0)	
T + A + P + MSI	1 (2.4)	1 (2.4)	0 (0)	
T + A + P	1 (2.4)	0 (0)	1 (2.4)	
T + P + MSI	1 (2.4)	0 (0)	1 (2.4)	
T + MSI	5 (11.9)	5 (11.9)	0 (0)	
A	1 (2.4)	0 (0)	1 (2.4)	
ECMO equipment				0.873
EBS	6 (14.3)	5 (11.9)	1 (2.4)	
PLS	36 (85.7)	29 (69)	7 (16.7)	

Abbreviations: *A* abdomen, *BMI* body mass index, *CPR* cardiopulmonary resuscitation, *ECMO* extracorporeal membrane oxygenation, *H* head, *IQR* interquartile range, *IS* inotropic score dopamine (mcg/kg/min) + dobutamine (mcg/kg/min) + 100 x epinephrine (mcg/kg/min) + 100, *MSI* musculoskeletal injury, *MV* mechanical ventilation, *P* pelvis, *SAPSII* Simplified Acute Physiology Score II, *SOFA* Sequential Organ Failure Assessment, *T* thorax, *VIS* Vasoactive-Inotropic Score IS + 100 x norepinephrine(mcg/kg/min) + 10 x milrinone (mcg/kg/min) + 10,000 x vasopressin (Units/kg/min)

*Statistically significant, *P* < 0.05

**Table 2:** Comparisons of laboratory findings between survivors and non-survivors

Pre-ECMO	Total	Survivors	Non-survivors	*P*
Variables, median	*n* = 42 (IQR)	*n* = 34 (IQR)	*n* = 8 (IQR)	
Hemoglobin (g/dL)	12.8 (10.6–15.3)	13.2 (10.8–15.6)	11.5 (8.0–12.6)	0.098
Platelet count (cells/μL)	199.5 (100.5–276.2)	237 (132.0–287.2)	79 (43.5–167.5)	0.003*
Activated PTT (s)	43.3 (31.5–64.1)	39.8 (30.8–50.0)	91.7 (62.6–107.4)	0.003*
INR	1.2 (1.1–1.4)	1.1 (1.0–1.3)	1.7 (1.7–2.5)	0.002*
ABGA				
pH	7.19 (7.01–7.31)	7.24 (7.0–7.3)	6.95 (6.83–7.06)	0.002*
PaO_2_ mmHg	60.5 (51.1–77.2)	60.5 (51.1–74.4)	58.7 (39.5–88.1)	0.144
PaCO_2_ mmHg	47.0 (38.9–62.4)	46.1 (38.5–59.0)	60 (50.3–109.7)	0.409
PaO_2_/FiO_2_	61.5 (49.7–81.7)	63 (51.1–80.7)	55.9 (35.0–83.8)	0.686
BUN (mg/dL)	15.7 (12.0–21.1)	15.7 (11.0–19.9)	16.8 (13.7–23.3)	0.519
Creatinine (mg/dL)	1.1 (0.8–1.3)	1.0 (0.8–1.3)	1.3 (0.8–1.9)	0.125
Bilirubin (mg/dL)	0.8 (0.4–1.6)	0.8 (0.4–1.6)	0.6 (0.2–1.8)	0.647
Lactate (mmol/L)	6.7 (2.6–11.5)	5.7 (2.4–9.2)	15.0 (12.5–18.4)	0.001*

Abbreviations: *ABGA* arterial blood gas analysis, *BUN* blood urea nitrogen, *ECMO* extracorporeal membrane oxygenation, *INR* international normalized ratio, *IQR* interquartile range, *PaCO2* partial arterial carbon dioxide pressure, *PaO2* partial arterial oxygen saturation, *PTT* partial thromboplastin time

*Statistically significant, *P* < 0.05

Patients received the following types of ECMO: VV ECMO, 29 (69%); VA, 7 (16.7%), among whom 1 (2.4%) subsequently received VV; VV followed by VAV, 2 (4.8%); VAV followed by VV, 1 (2.4%) and VAV, 3 (7.1%). The median time of door to ECMO was all 3.5 h for total, survivors, and non-survivors group (*p* = 0.328). The number of patients who received ECMO within 24 h was 28, 23, and 5 for total, survivors, and non-survivors group (*p* = 0.543). The median ICU stay was 16 days. Thirty-four of 42 patients (81%) survived and were discharged after a median hospital stay of 23 days (Table 3). Patients receiving VV ECMO had better survival rates than those receiving other types of ECMO.

**Table 3:** Comparison of treatments and outcomes between survivors and non-survivors

	Total	Survivors	Non-survivors	*P*
	*n* = 42 (%)	*n* = 34 (81%)	*n* = 8 (19%)	
ECMO types				0.001*
VV	29 (69)	28 (66.7)	1 (2.4)	
Other types	13 (31)	6 (14.3)	7 (16.7)	
Anticoagulation				0.655
Heparin	11 (26.2)	9 (21.4)	2 (4.8)	
Nafamostat mesilate	31 (73.8)	25 (59.5)	6 (14.3)	
ECMO time (hours), median (IQR)	155 (90.5–232.5)	160 (114.0–240.7)	24 (4.0–132.0)	0.021*
CRRT	24 (57.1)	19 (45.2)	5 (11.9)	0.527
Transfusion (U/day) during ECMO period, median (IQR)				
Packed RBC	1 (0.5–2.6)	0.7 (0.4–1.7)	5.7 (1.6–59.2)	0.001*
Fresh frozen plasma	0.2 (0–1.3)	0.2 (0–0.7)	4.7 (0.9–29.3)	0.001*
Platelet concentrate	0.1 (0–2.3)	0 (0–1.4)	7 (0–11.5)	0.001*
Cryoprecipitate	0.0 (0–0)	0.0 (0–0)	0 (0–2.9)	0.09
ICU stay (days), median (IQR)	16 (7.7–24.2)	17.5 (9.7–25.7)	3 (1.0–8.5)	0.014*
Hospital stay (days), median (IQR)	23 (13.2–51.2)	28 (18–58.5)	3 (1.0–8.5)	0.006*

Abbreviations: *CRRT* continuous renal replacement therapy, *ECMO* extracorporeal membrane oxygenation, *ICU* intensive care unit, *IQR* interquartile range, *RBC* red blood cells, *VV* veno-venous

*Statistically significant, *P* < 0.05

### Comparisons between survivors and non-survivors

Next, we compared the characteristics of survivors and non-survivors to identify risk factors for mortality. Notably, survivors had a lower VIS and SAPSII (Table 1) and higher platelet count and arterial blood pH, while non-survivors had higher prolonged aPTT, INR and lactate concentration values (Table 2). Survivors also required significantly fewer transfusions (Table 3). The following complications occurred during ECMO: acute renal failure (creatinine > 2 mg/dL) in 11 (26.2%) patients, bed sores in 2 (4.8%), cholecystitis in 2 (4.8%), multi-organ failure in 2 (4.8%), central nervous system injury (hemorrhage) in 1 (2.4%), ulcer bleeding in 1 (2.4%), and leg ischemia in 1 (2.4%) patient (Table 4).

**Table 4:** Comparison of complications during ECMO between survivors and non-survivors

	Total	Survivors	Non-survivors	*P*
	*n* = 42 (%)	*n* = 34 (81%)	*n* = 8 (19%)	
Leg ischemia	1 (2.4)	0 (0)	1 (2.4)	0.190
Bed sore	2 (4.8)	2 (4.8)	0 (0)	0.652
ARF (creatinine > 2 mg/dL)	11 (26.2)	5 (11.9)	6 (14.3)	0.002*
Cholecystitis	2 (4.8)	2 (4.8)	0 (0)	0.652
Ulcer bleeding	1 (2.4)	1 (2.4)	0 (0)	0.810
CNS injury	1 (2.4)	0 (0)	1 (2.4)	0.190
Multiorgan failure	2 (4.8)	1 (2.4)	1 (2.4)	0.348

Abbreviations: *ARF* acute renal failure, *CNS* central nervous system, *ECMO* extracorporeal membrane oxygenation

*Statistically significant, *P* < 0.05

**Fig. 2:**
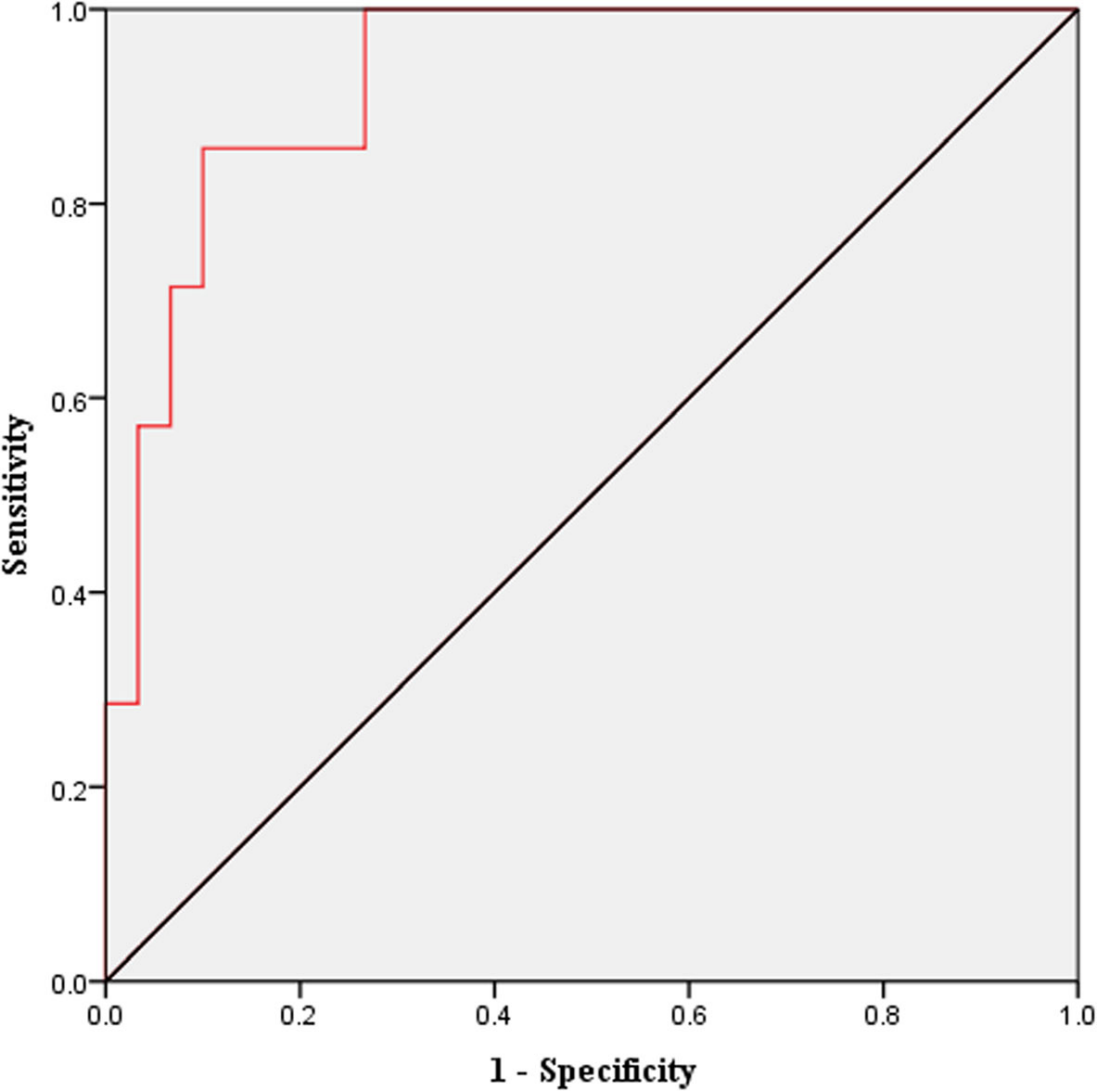
Receiver operating characteristic curve for predictability of pre-ECMO lactate level for survival to hospital discharge. The area under the receiver operating characteristic curve was 0.929 (95% confidence interval: 0.840 to 1.000, *P* = 0.001) for pre-ECMO lactate level. The optimal cut off value of pre-ECMO lactate level was 10.5 mmol/L for predicting survival at hospital discharge. In analysis of pre-ECMO lactate level 10.5 mmol/L, sensitivity of 85.7%, specificity of 86.7%, positive predictive value of 93.8%, and negative predictive value of 60.0% were noted. Abbreviation: ECMO, extracorporeal membrane oxygenation.

**Fig. 3:**
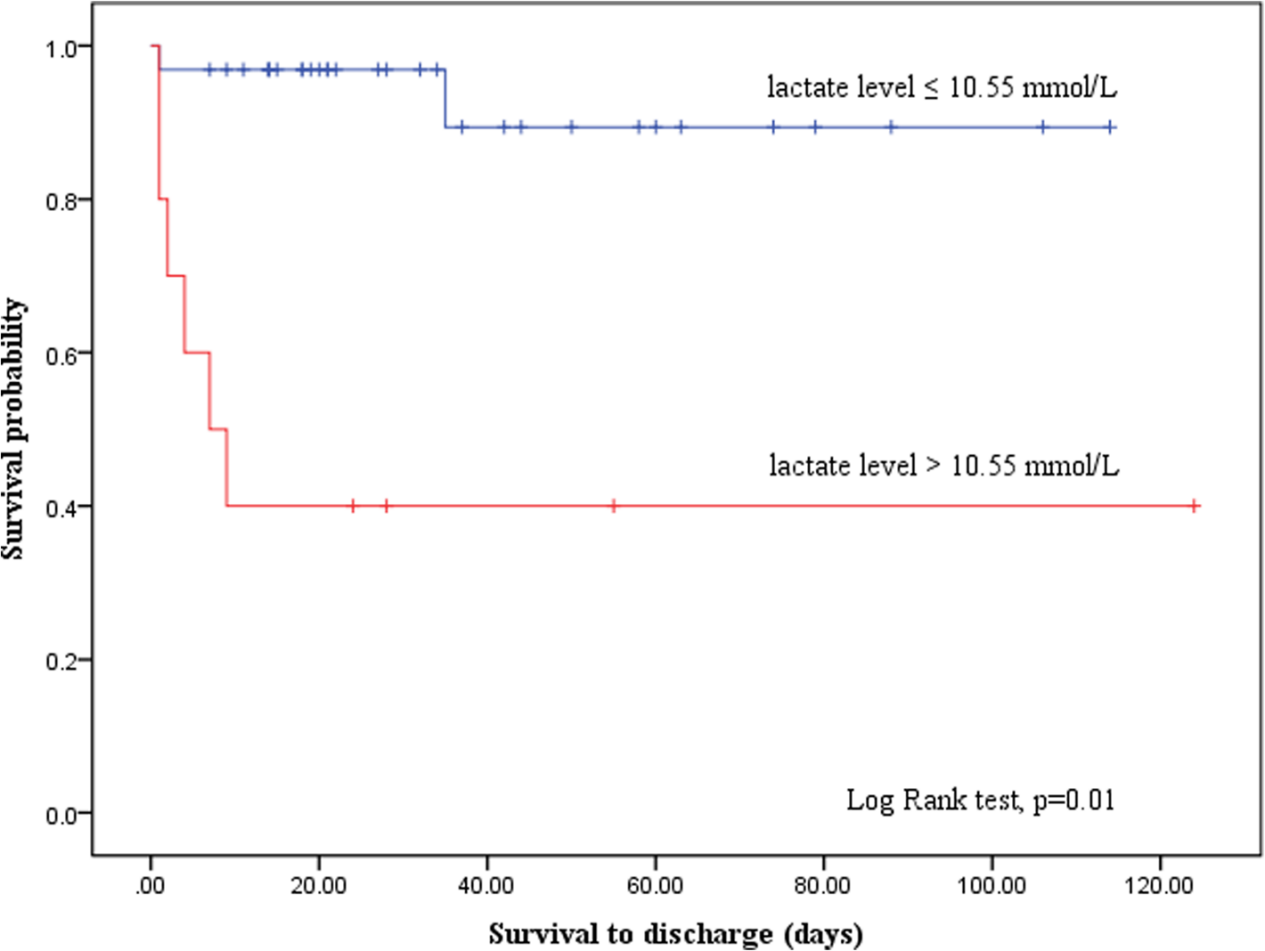
Kaplan-Meier survival analysis based on lactate concentration. Kaplan–Meier survival curve for survival to hospital discharge between patients with pre-ECMO lactate level above 10.5 mmol/L(red line) and below 10.5 mol/L(blue line). The cumulative survival rate at hospital discharge was significantly higher for patients with a pre-ECMO lactate level of 10.5 mmol/L or less(blue line) compared with patients having a pre-ECMO lactate level greater than 10.5 mmol/L(red line, 93.8% versus 40.0%, respectively; *P =* 0.01)

The investigated pre-ECMO variables were defined by considering the overlapping meaning between the variables. Ultimately, we included six variables identified as significant in the univariate analysis—SAPSII, aPTT, INR, pH, platelet count and lactate level—in a subsequent multivariate analysis (Table 5). We identified the lactate level as an independent predictor of survival [odds ratio (OR), 1.493; 95% confidence interval (CI), 1.060–2.103, *P =* 0.022], with an area under the receiver operating characteristic curve of 0.929 (95% CI, 0.840–1.000, *P =* 0.001; Fig. 2) and estimated cut-off value of 10.5 mmol/L (sensitivity: 0.857, specificity: 0.867). This cut-off yielded a significant difference in survival in a Kaplan–Meier analysis (*P* = 0.01, Fig. 3). Finally, univariate and multivariate analyses of four treatment variables revealed a favorable association of VV ECMO (OR: 0.075, 95% CI: 0.006–0.901, *P* = 0.041) with survival (Table 6).

**Table 5:** Associations of pre-ECMO variables with survival in univariate and multivariate analyses

	Univariate		*P*	Multivariate		*P*
	Odds ratio	95% CI		Odds ratio	95% CI	
SAPSII score	1.068	1.010–1.129	0.022	–	–	–
aPTT	1.031	1.006–1.057	0.014	–	–	–
INR	4.610	1.378–15.417	0.013	–	–	–
pH in ABGA	0.001	0.000–0.163	0.009	–	–	–
Platelet count	0.984	0.972–0.997	0.013	0.978	0.956–1.001	0.063
Lactate concentration	1.536	1.131–2.087	0.006	1.493	1.060–2.103	0.022*

Abbreviations: *ABGA* arterial blood gas analysis, *CI* confidence interval, *ECMO* extracorporeal membrane oxygenation, *INR* international normalized ratio, *PTT* partial thromboplastin time, *SAPSII* Simplified Acute Physiology Score II, *VIS* vasoactive inotropic score

*Statistically significant, *P* < 0.05

**Table 6:** Associations of treatment variables with survival in univariate and multivariate analyses

	Univariate		*P*	Multivariate		*PP*
	Odds ratio	95% CI		Odds ratio	95% CI	
Packed RBC	1.993	1.113–3.568	0.020	–	–	–
Platelet concentration	1.388	1.090–1.768	0.008	–	–	–
Fresh frozen plasma	1.983	1.076–3.378	0.028	1.811	0.866–3.788	0.114
ECMO types	0.031	0.003–0.297	0.003	0.075	0.006–0.901	0.041*

Abbreviations: *CI* confidence interval, *ECMO* extracorporeal membrane oxygenation, *RBC* red blood cells

*Statistically significant, *P* < 0.05

## Discussion

Trauma patients with severe injuries are at risk of ARDS via direct and indirect causes, including lung contusions, aspiration pneumonia, massive blood transfusion and fat embolism syndrome [11]. ARDS remains a common post-traumatic complication and is diagnosed in 6.5% of patients requiring mechanical ventilation for > 48 h [12]. If optimal ventilator strategies and adjunctive measures fail to yield improvement, ECMO might be considered to provide temporary respiratory or cardiac support in patients with cardiopulmonary failure and to allow the lungs to rest through manipulation the ventilator settings and the concentration of inspired oxygen while maintaining appropriate oxygenation, carbon dioxide removal, and tissue perfusion [13]. However, this potentially life-saving intervention is not without complications. Bleeding and thrombotic complications are the major causes of ECMO-associated morbidity and mortality [14]. The use of anticoagulant therapy is recommended to prevent thrombosis during ECMO support, however, the risk of thromboembolic complications must be balanced with the risk of bleeding. Accordingly, the use of ECMO in trauma patients is limited by the high risk of hemorrhage during and after cannulation, particularly in the presence of severe coagulopathy, contraindications to anticoagulant treatment and the risk of intracranial hemorrhage following traumatic brain injury. However, technological advances have significantly improved the characterizations of bleeding profiles. Arlt et al. [15] reported that the delayed use of heparin in 10 trauma patients did not lead to any adverse thrombotic events. Moreover, a case series described 3 cases involving delayed heparin administration due to traumatic hemorrhage and/or brain injury [16], with times to heparinization after cannulation ranging from 24 h to 5 days. All the patients survived to discharge, and only 1 experienced a thrombotic complication (IVC clots) that was resolved by heparinization after decannulation.

We administered nafamostat mesilate during ECMO support to patients with acute renal failure or at a high risk of bleeding. This synthetic serine protease inhibitor has a very short half-life and inhibits coagulation by inactivating thrombin, the activated coagulation factors XIIa and Xa, complement factors C1r and C1s, plasmin, trypsin and kallikrein 10, 11, 12, 13 and 14. We previously used nafamostat instead of heparin to reduce bleeding complications in patients receiving ECMO, with acceptable results [7].

Regarding survival, Cordell-Smith et al. [13] reported that 20 of 28 patients (71%) who received ECMO for severe trauma-related respiratory failure survived, with pre-ECMO ventilation times of 61 and 87 h for survivors and non-survivors, respectively. Ried et al. [17] evaluated 26 patients who received VV ECMO for severe trauma-related respiratory failure after mean 2.6 days of pre-ECMO ventilation and reported an 81% survival rate. Another study reported an overall survival rate of 56% among 176 patients supported with VV ECMO, and the best survival rate among trauma patients with a pre-ECMO ventilation interval of 4 days (71%, 10/14 patients) [18]. Similarly, we calculated an overall survival rate of 81% (*n* = 34) in our cohort of 42 patients, 29 (69%) of whom were supported with VV ECMO. Both survivors and non-survivors received pre-ECMO ventilation for 1 day. We observed associations of the pre-ECMO VIS and SAPSII with mortality during ECMO support, consistent with our previous observation of the former as an important predictor of mortality in patients receiving ECMO support in emergency departments [6]. Therefore, we suggest that a preexisting severity scoring system may be useful for predicting mortality in trauma patients. However, the SOFA score had limited utility in our study population.

Regarding survival predictors, Enger and colleagues [19] developed a new lactate-based risk score for pre-ECMO mortality and reported significantly higher values in non-survivors than in survivors. In our multivariate analysis, we similarly identified lactate level as a significant predictor of survival, using a cut-off value of 10.5 mmol/L.

Generally, VV ECMO assists the lungs, VA ECMO assists the heart, and VAV ECMO simultaneously assists the lungs and heart. In the patients with acute respiratory failure due to traumatic lung injury or posttraumatic ARDS, the VV ECMO application is the most common due to hypoxia and accounted for 69% (*n* = 29) in this present study. In other cases, VA ECMO (*n* = 7, 16.7%) was applied when the circulatory failure was main problem than hypoxia, and VAV ECMO (*n* = 6, 14.3%) was applied when both the lungs and the heart was main problem and needed assistance at the same time. It is commonly known that the mortality increases when two organs are involved rather than one. Furthermore, the 2020 Extracorporeal Life Support Organization Registry reported that the rate of survival to discharge in the patients with pulmonary support was higher than those with cardiac support or extracorporeal cardiopulmonary resuscitation (60% versus 43 and 29%). Similarly, VV ECMO was also associated with better survival outcomes than other types of ECMO support in the present study. We attribute the reasons of these results to be high severity of injuries and hazard statuses of patients receiving other types of ECMO, who usually require cardiac and pulmonary support. Guirand et al. [20] reported an independent association between VV ECMO support and survival in adult trauma patients, regardless of the transfusion requirement or complications due to the frequency of bleeding. Similarly, VV ECMO was also associated with better survival outcomes vs. other types of ECMO support. We attribute this finding to the severity of injuries and hazard statuses of patients receiving other types of ECMO, who usually require cardiac and pulmonary support.

Our study had some limitations. First, the cohort was small, uncontrolled, unicenter and the retrospective design may have introduced unidentified bias. Furthermore, we did not investigate long-term follow-up data. Although ECMO remains controversial in trauma patients with severe ARDS, its increased use suggests nationwide acceptance and/or increased availability at trauma centers. Our observation that ECMO support is associated with favorable outcomes in patients with traumatic ARDS suggests the validity of this salvage method in this population.

## Conclusions

We observed a good survival rate among patients receiving ECMO following acute respiratory failure due to severe traumatic injury. Lung support ECMO may be a feasible, safe, and favorable alternative to conventional treatment in a carefully selected trauma population.

## Data Availability

The data that support the finings of this study are available from the corresponding author upon reasonable request.

## Abbreviations

ARDS: Acute respiratory distress syndrome
ECMO: Extracorporeal membrane oxygenation
VV-ECMO: Veno-venous extracorporeal membrane oxygenation
PaO2: Partial arterial oxygen pressure
FiO2: Fractional inspired oxygen concentration
VA ECMO: Venoarterial extracorporeal membrane oxygenation
V-AV ECMO: Venoarteriovenous extracorporeal membrane oxygenation
SaO2: Oxygen saturation
aPTT: Activated partial thromboplastin time
CRRT: Continuous renal replacement therapy
RBC: Red blood cells
FFP: Fresh frozen plasma
INR: International normalized ratio
VIS: Vasoactive-Inotropic Score
SAPSII: Simplified Acute Physiology Score II
SOFA: Sequential Organ Failure Assessment
IQR: Interquartile range
ANOVA: Analysis of variance
CI: Confidence interval
